# Estimating Cardiorespiratory Fitness Without Exercise Testing or Physical Activity Status in Healthy Adults: Regression Model Development and Validation

**DOI:** 10.2196/34717

**Published:** 2022-07-06

**Authors:** Robert Sloan, Marco Visentini-Scarzanella, Susumu Sawada, Xuemei Sui, Jonathan Myers

**Affiliations:** 1 Department of Social and Behavioral Medicine Kagoshima University Graduate Medical School Kagoshima Japan; 2 Faculty of Sport Sciences Waseda University Saitama Japan; 3 Department of Exercise Science Arnold School of Public Health University of South Carolina Columbia, SC United States; 4 Division of Cardiovascular Medicine Veterans Affairs Palo Alto Health Care System Stanford University Palo Alto, CA United States

**Keywords:** nonexercise estimated cardiorespiratory fitness, public health, surveillance, epidemiology, electronic health record, EHR, fitness, cardiorespiratory, physical activity, regression model, nonexercise equation

## Abstract

**Background:**

Low cardiorespiratory fitness (CRF) is an independent predictor of morbidity and mortality. Most health care settings use some type of electronic health record (EHR) system. However, many EHRs do not have CRF or physical activity data collected, thereby limiting the types of investigations and analyses that can be done.

**Objective:**

This study aims to develop a nonexercise equation to estimate and classify CRF (in metabolic equivalent tasks) using variables commonly available in EHRs.

**Methods:**

Participants were 42,676 healthy adults (female participants: n=9146, 21.4%) from the Aerobics Center Longitudinal Study examined from 1974 to 2005. The nonexercise estimated CRF was based on sex, age, measured BMI, measured resting heart rate, measured resting blood pressure, and smoking status. A maximal treadmill test measured CRF.

**Results:**

After conducting nonlinear feature augmentation, separate linear regression models were used for male and female participants to calculate correlation and regression coefficients. Cross-classification of actual and estimated CRF was performed using low CRF categories (lowest quintile, lowest quartile, and lowest tertile). The multiple correlation coefficient (*R*) was 0.70 (mean deviation 1.33) for male participants and 0.65 (mean deviation 1.23) for female participants. The models explained 48.4% (SE estimate 1.70) and 41.9% (SE estimate 1.56) of the variance in CRF for male and female participants, respectively. Correct category classification for low CRF (lowest tertile) was found in 77.2% (n=25,885) of male participants and 74.9% (n=6,850) of female participants.

**Conclusions:**

The regression models developed in this study provided useful estimation and classification of CRF in a large population of male and female participants. The models may provide a practical method for estimating CRF derived from EHRs for population health research.

## Introduction

### Background

The use of data from electronic health records (EHRs) beyond day-to-day medical management is rapidly emerging in the fields of digital health, public health, and epidemiology [1,2]. However, access to cardiorespiratory fitness (CRF), a valuable health metric, is limited. This limitation is primarily due to the medical service (cardiopulmonary stress test) being costly, time-consuming, and generally focused on cardiac patients [3-5]. CRF is a comprehensive measure of one’s functional capacity (mL O_2_ · kg^‒1^ · min^‒1^) driven by the combination of heart, lung, and muscle function [6]. It is an important marker of health status in the general adult population [3,6]. Further demonstrating the importance of CRF, the American Heart Association released a scientific statement proposing that CRF be considered a clinical vital sign. The scientific rationale behind the statement is driven by the voluminous evidence that demonstrates that low CRF is a strong independent predictor of adverse health outcomes (ie, all-cause mortality, cancer, stroke, heart disease, and diabetes incidence) [3].

### Prior Work

To help increase accessibility to CRF data, researchers have developed an array of nonexercise estimated CRF (NEECRF) equations to estimate CRF [7-9]. NEECRF equations commonly include age, gender, resting heart rate, smoking status, BMI, and self-reported physical activity status (PAS) [8,10-12]. Studies have shown NEECRF to predict all-cause and cardiovascular disease mortality on par with measured CRF [13,14]. However, the assessment of PAS required to calculate NEECRF in patients is not typically conducted or documented in health care settings [15]. Therefore, using an NEECRF model without PAS (non-PAS) may be more feasible.

In a 2019 comprehensive NEECRF review, a few peer-reviewed non-PAS NEECRF equations were identified in adult populations reporting correlations and SE estimates [8]. Most of the equations were developed using only age, height, and weight combinations, and some used variables not commonly found in EHRs (eg, waist girth, predicted/ideal weight, or exercise mode).

Though correlations were moderate to high, the sample populations were too small to determine the classification accuracy of low CRF. Accurate classification of low CRF is essential for large-scale investigations [3,16]. Low CRF (lowest tertile) classification was recently investigated by Peterman et al [9], who found Baynard’s [7] simplified non-PAS NEECRF equation to have poor classification ability in a sizable (n=4871) adult population. Other researchers have also suggested that simple non-PAS NEECRF formulas have low validity, and nuanced approaches are warranted [16,17]. Investigators have also noted that valid non-PAS NEECRF models may have broad applications for public health, epidemiology, surveillance, practice, and research [3,8,9,16,18].

### Goal of This Study

Because it is standard practice to assess and document resting heart rate, blood pressure, BMI, and smoking status during a typical clinic visit, the primary aim of this study was to develop new models for NEECRF that could potentially be used in large-scale population health investigations using variables commonly found in EHRs. To accomplish this, we compared a non-PAS NEECRF equation to clinically measured CRF and evaluated its ability to estimate and classify CRF.

## Methods

### Study Sample

The Aerobics Center Longitudinal Study (ACLS) is a prospective epidemiological investigation of participants that began in 1970 [19]. The original data set for this investigation included 43,257 healthy adults who voluntarily participated in a comprehensive preventive medical examination at the Cooper Clinic in Dallas, Texas between 1974 and 2005. At baseline, all participants were free of diabetes, heart disease, stroke, cancer, positive electrocardiograms, and completed a maximal graded exercise test. Each participant gave informed consent to join the longitudinal study. The research population demographic primarily consists of Caucasian college-educated adults of middle to high socioeconomic status with an average age at baseline of 43.5 (range 20-79) years.

### Ethical Approval

The study was reviewed and approved annually by the Cooper Institute Institutional Review Board, and all participants provided written informed consent.

### Measurements

Predictor variables were assessed during a preventive health examination that included objective measurements of age, BMI, resting heart rate, systolic blood pressure, diastolic blood pressure, and self-reported smoking status between 1974 and 2005 at the Cooper Clinic. Age was verified at the time of the examination. Height and weight were measured on a calibrated scale using US customary units and converted to metric scales for this investigation. BMI was calculated from measured height and weight as kg/m^2^. Manual auscultation was used to measure resting blood pressure while seated. Resting heart rate was calculated using the R-R interval on an electrocardiogram while seated. CRF was expressed as absolute metabolic equivalent tasks (METs; 1 MET = 3.5 mL O_2_ · kg^‒1^ · min^‒1^) based on the total duration of a symptom-limited maximal Balke graded exercise test [6]. Following American College for Sports Medicine Guidelines, patients were encouraged to give maximal effort, and the test end point was volitional exhaustion or termination by the physician for medical reasons [6]. METs were calculated based on the final treadmill speed and grade [6]. The Balke graded exercise test is highly correlated (*r*=0.94) with maximal graded cardiopulmonary exercise testing [20,21]. A standardized medical questionnaire was used to ascertain demographic information, lifestyle habits, and chronic disease status. More detailed information on the preventive health examination is available in prior ACLS publications [10,13].

### Statistical Analysis

#### Data Exclusion

Data were first examined for outliers and skewness. We removed the outliers from the data set by removing participants with predictor variables (continuous) beyond the ±3 σ interval. To do this, we calculated the mean and SD of each predictor variable, excluding smoking (categorical). Any participant with at least one predictor variable above 3 SDs or below 3 SDs was flagged as an outlier. After removing outliers and incomplete entries (n=581), the data set comprised 33,530 male participants and 9146 female participants, 98.7% of the original 43,257 participants.

#### Regression

The main analysis was based on apparently healthy adults at baseline. Using a supervised machine learning technique, we conducted separate linear regression analyses for men and women to predict non-PAS NEECRF based on nonlinear augmentation of the predictor variables [1]. We also considered advanced machine learning models but did not find them advantageous. The male and female non-PAS NEECRF prediction equations were formulated to minimize the average mean squared error, where *N* is the number of samples in our data set:



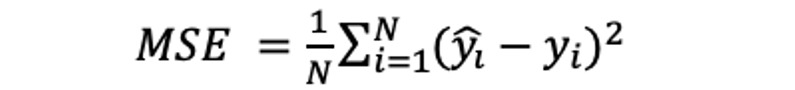



The prediction equation used age, height (Ht), weight (Wt), BMI, resting heart rate (rHR), systolic blood pressure (SBP), diastolic blood pressure (DBP), and smoking. All variables were continuous except for smoking status (nonsmoker=0, current smoker=1). Data were standardized by subtracting the mean and dividing by the SD for each variable. Next, separate models were then trained for male and female participants. We augmented the original 8 variables with second order and interaction terms, and regressed them linearly to the dependent variable for training. In this way, the nonlinearity was transferred from the regressor to the independent variables, while the model’s overall interpretability was maintained. The augmentation procedure added the following 28 second order and interaction terms: Wt^2^, Wt × Ht, Wt × Age, Wt × rHR, Wt × SBP, Wt × DBP, Wt × BMI, Ht^2^, Ht × Age, Ht × rHR, Ht × SBP, Ht × DBP, Ht × BMI, Age^2^, Age × rHR, Age × SBP, Age × DBP, Age × BMI, rHR^2^, Ht × SBP, Ht × DBP, rHR × BMI, SBP^2^, SBP × DBP, SBP × BMI, DBP^2^, and DBP × BMI (for a total of 36 variables). Because smoking status was a categorical variable, it was not used to create the additional variables. The augmented data set was input into an elastic net linear regressor and trained and evaluated via 10-fold cross-validation [22]. Optimal model hyperparameters were calculated for the male (α=.001, λ=1.0) and female (α=.004, λ=1.0) data sets through the cross-validation procedure. Pearson correlation coefficients were then calculated using the non-PAS NEECRF equations for the male and female data sets (shown in Multimedia Appendix 1). Lastly, for comparison, we cross-validated Baynard’s [7] simplified non-PAS NEECRF equation (77.96 − 10.35 (sex; M=0, F=1) − 0.92 (BMI) − 0.32 (age)) [9].

#### Classification Accuracy

We cross-classified non-PAS NEECRF and CRF for three specified cut points (lowest quintile, lowest quartile, and lowest tertile) commonly used in epidemiological investigations to define low CRF [3]. Next, CRF distributions were then grouped by males and females where α served as the value of the α-th percentile using the calculation non-PAS NEECRF>α. After classification, we determined the non-PAS NEECRF accuracy, sensitivity, positive predictive value, and *F*_1_ score. The reference standard was the measured CRF, and low CRF was defined as a positive test. All analyses were performed in scikit-learn version 0.22.2 (NumFOCUS).

## Results

Descriptive statistics are provided in Table 1. Correlation coefficients between each independent variable and CRF are presented in Multimedia Appendix 1. The multiple Rs and mean deviations for non-PAS NEECRF (in METs) were high at 0.70 (mean deviation 1.33) for male participants and moderate at 0.65 (mean deviation 1.23) for female participants. The models explained 48.4% (SE estimate 1.70, 95% CI 0.05-3.97) of the variance in CRF for male participants and 41.9% (SE estimate 1.56, 95% CI 0.05-3.48) for female participants. Multimedia Appendix 1 provides a simple independent variable input Google Sheet for researchers and data scientists to easily calculate NEECRF. Table 2 provides the findings regarding the accuracy, positive predictive, and sensitivity values using the lowest quintile, quartile, and tertile to classify low CRF for male and female participants. While overall classification accuracy was meaningful for a nondiagnostic test across the three models, the optimal model was the lowest tertile. Combined male and female positive predictive value were 0.60, sensitivity 0.67, and *F*_1_ score 0.63 [23,24]. The *F*_1_ score is the best practice summary metric consisting of the harmonic mean of positive predictive value and sensitivity for classification (0=low, 1=high) [23]. Assuming a balanced data set (n=2529) by Peterman et al [16], we calculated the *F*_1_ scores from their reported findings for equations applicable to EHRs [16]. We found *F*_1_ scores ranging from 0.04 to 0.56. Based on a residual plot (Multimedia Appendix 1), we found the model was most accurate for CRF MET values in the 7.5 to 12.5 METs range but tended to underestimate MET values >12.5 METs and overestimate MET values <7.5 METs. Notably, this is a common finding in non-PAS NEECRF studies [3]. Similar to Peterman et al [9], we cross-validated Baynard’s [7] non-PAS NEECRF equation with our reference CRF data set and found low positive correlations for male (*r*=0.49, mean deviation 1.60) and female (*r*=0.43, mean deviation 1.46) participants.

**Table 1:** Baseline characteristics of participants.

	All (N=42,676)	Male participants (n=33,530)	Female participants (n=9146)
Age (years), mean (SD)	44.1 (9.6)	44.1 (9.5)	44.1 (10.2)
BMI (kg/m^2^), mean (SD)	25.8 (3.8)	26.5 (3.5)	23.3 (3.7)
Resting heart rate (bpm), mean (SD)	60.9 (10.5)	60.1 (10.4)	64.0 (10.1)
Systolic blood pressure (mmHg), mean (SD)	118.9 (13.4)	120.7 (12.7)	112.4 (13.9)
Diastolic blood pressure (mmHg), mean (SD)	80.0 (9.6)	81.1 (9.3)	76.0 (9.3)
Smoker, n (%)	6361 (14.9)	5569 (16.7)	792 (9.7)
Measured CRF^a^ (mL/kg/min), mean (SD)	11.5 (2.5)	12.0 (2.4)	9.8 (2.0)

^a^CRF: cardiorespiratory fitness (maximal oxygen consumption).

**Table 2:** Predictive accuracy of nonexercise estimated CRF classification of lowest cardiovascular fitness against the reference CRF.

Group	Low CRF^a^ (lowest quintile)				Low CRF (lowest quartile)				Low CRF (lowest tertile)		
	ACC^b^ (%)	PPV^c^ (%)	SEN^d^ (%)	ACC (%)		PPV (%)	SEN (%)	ACC (%)		PPV (%)	SEN (%)
Male participants	81.5	44.7	69.2	79.5		50.9	67.4	77.2		59.9	66.7
Female participants	80.6	43.9	57.8	77.9		55.4	55.6	74.9		60.4	66.9

^a^CRF: cardiorespiratory fitness.

^b^ACC: accuracy.

^c^PPV: positive predictive value.

^d^SEN: sensitivity.

## Discussion

### Principal Findings

Using a greater combination of clinical measures commonly found in EHRs, this study compared non-PAS NEECRF with objectively measured CRF in the largest population to date [10,11,25-27]. Overall, our model may provide a more applicable method for estimating and classifying CRF than previous methods [8,9,16]. Moreover, because the vital signs and medical information used to calculate non-PAS NEECRF are routinely captured during health care visits, our approach places nominal demand on health care staff and patients for collecting data. From a public health perspective, a moderate positive predictive value is practical, given that non-PAS NEECRF is a nondiagnostic test that is no cost and easily accessible [24]. Likely, some individuals classified with low fitness may be at the lower end of the fit spectrum and benefit from health promotion [24]. From a clinical perspective and considering moderate sensitivity, we concur with previous investigators that, while estimation equations are applicable for epidemiological investigations, they should not replace clinical exercise testing for patient diagnosis and management [3,16]. Our findings show that the ACLS non-PAS NEECRF may provide a useful assessment of CRF to conduct population health research.

### Comparison to Prior Work

Comparatively, PAS-based NEECRF models have demonstrated higher positive correlation values (0.71-0.93) along with a higher degree (~90%) of correct classification accuracy for low CRF than non-PAS NEECRF models [3]. Because physical activity is a key contributor to CRF, including a PAS variable in an NEECRF model improves accuracy [3,8,16]. However, a recent review of distinct EHRs across 20 countries found that only 18.8% of family practice clinics had structured PAS questionaries embedded within the EHR, with documented PAS in the EHR ranging from 10% to 86% [15]. Notably, no validated questionaries designed for PAS-based NEECRF calculations were used. Therefore, the ability to conduct large-scale studies aggregating existing EHR data across local, domestic, or international systems to predict CRF is unlikely. Conversely, our model may provide a global approach to aggregating EHR data across systems to predict CRF and conduct analyses.

In 2019, Wang et al [8] provided a comprehensive list of peer-reviewed non-PAS NEECRF models that used some combination of age, BMI, or gender to predict CRF [8]. Samples were from small populations, and moderate to high correlations were reported generally without SE estimates [8]. Notably, the validity and usefulness of these simplified types of equations have been called into question [8,9,16,17]. We found that the findings from such investigations may be limited because the studies only reported correlations and lacked sufficient sample sizes to calculate prediction values for low CRF. It is also important to note that high correlation values do not necessarily result in a more accurate classification of low CRF [9,16].

Recently, Peterman et al [16] determined the ability of 7 non-PAS NEECRF equations to accurately classify low NEECRF (tertile) compared to measured CRF in a demographically comparable cohort (n=2529) to ACLS. Only 3 of the 7 equations apply because of variables not commonly found in EHRs. On balance, the classification accuracy of low CRF (tertile) appeared to be better for ACLS. In a separate investigation, Peterman et al [9] also tested Baynard’s [7] simplified non-PAS NEECRF using the Ball State cohort (n=4871) to assess classification accuracy. The equation had an *r* of 0.76; however, there was poor accuracy for detecting individuals positive for low CRF (37%). We also cross-validated Baynard’s [7] equation with our data set and found low correlations, thus did not attempt to determine accuracy [9]. Although the ACLS and Ball State cohorts are demographically similar, our finding is expected to some degree because the ACLS equation is specifically trained from the ACLS data set [9,16]. Moreover, these equations need to be tested in epidemiological investigations with EHR data to see how well they predict health outcomes.

### Limitations

This study is not without limitations. Our study’s primary limitation regarding correlation was that the measured reference CRF was conducted using a Balke graded maximal exercise test that estimates absolute METs. This testing strongly correlates with adults’ maximal graded cardiopulmonary exercise testing and is routinely used for clinical and epidemiological purposes [3,20,21]. Though the ACLS data set provides the largest known healthy female data set for clinically measured CRF, a larger female sample size may have provided a slightly better predictive model [3,27]. Nonetheless, our analyses demonstrated reasonable correlation and classification. Our analyses are based on a large predominantly Caucasian cohort; it is unknown if the results generalize to other ethnic groups. Notably, the homogeneity of the ACLS cohort may have strengthened the internal validity of our results by limiting possible confounders. The main strength of this investigation is that it was conducted on the largest cohort to date with a larger number of objectively measured predictive variables to estimate non-PAS NEECRF.

### Conclusions

The ACLS non-PAS NEECRF equation may provide a useful population health metric for CRF. More work should be conducted regarding diverse populations, the incidence of chronic conditions, and longitudinal repeated measures analyses toward improving public health and surveillance capability.

## Appendix

Multimedia Appendix 1 Supplementary material.

## Abbreviations

ACLS: Aerobics Center Longitudinal Study
CRF: cardiorespiratory fitness
DBP: diastolic blood pressure
EHR: electronic health record
Ht: height
MET: metabolic equivalent task
NEECRF: nonexercise estimated cardiorespiratory fitness
PAS: physical activity status
rHR: resting heart rate
SBP: systolic blood pressure
Wt: weight
