# Supplementary material for: Estimating Cardiorespiratory Fitness Without Exercise Testing or Physical Activity Status in Healthy Adults: Regression Model Development and Validation
Source: JMIR Public Health Surveill. 2022 Jul 6;8(7):e34717. doi: 10.2196/34717 (PMC9301546; doi:10.2196/34717)
Supplement: Multimedia Appendix 1 [file publichealth_v8i7e34717_app1.pdf]

Supplement 1

Correlations between independent variables and CRF

**Male**		**Female**	
Variable	Pearson's coefficient	Variable	Pearson's coefficient
**Age**<br>**SBP**<br>**DBP**<br>**Resting Heart Rate**<br>**BMI**<br>**Smoker**	**-.34**<br>**-.15**<br>**-.22**<br>**-.43**<br>**-.49**<br>**-.32**	**Age**<br>**SBP**<br>**DBP**<br>**Resting Heart Rate**<br>**BMI**<br>**Smoker**	**-.39**<br>**-.22**<br>**-.20**<br>**-.35**<br>**-.44**<br>**-.14**

Residual plot

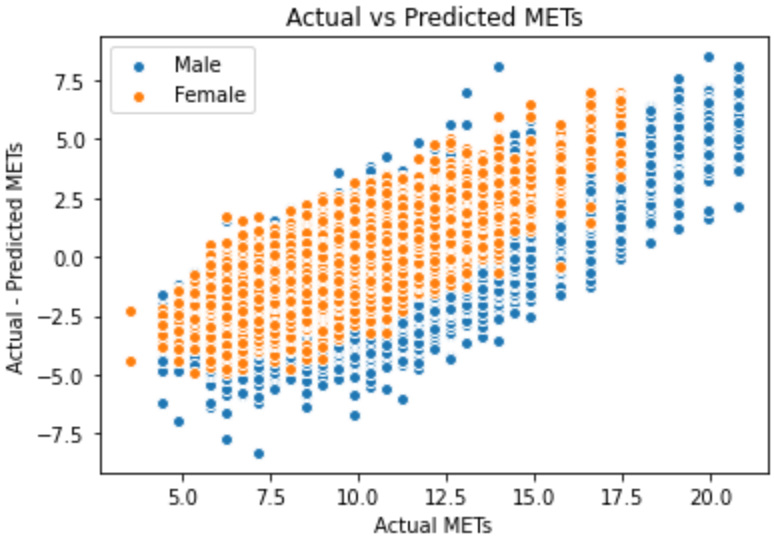

Google sheet link:
https://docs.google.com/spreadsheets/d/1lnYl4u62AMSUxkzyzKsKBHe8vr3WzLVRYxGyYE7xoGQ/edit#gid=1558519083

Men's equation

$$
\begin{aligned}
\mathrm{METs} = {} & -0.70\left(\frac{Age-44.14}{9.49}\right)+0.04\left(\frac{Ht-179.15}{6.51}\right)-0.85\left(\frac{rHR-60.10}{10.45}\right)-0.96\left(\frac{BMI-26.46}{3.50}\right)\\
& +0.24\left(\frac{SBP-120.58}{12.70}\right)-0.08\left(\frac{DBP-81.09}{9.32}\right)-0.14\left(\frac{Age-44.14}{9.49}\right)^2\\
& -0.05\left(\frac{Age-44.14}{9.49}\right)\left(\frac{Ht-179.15}{6.51}\right)+0.02\left(\frac{Age-44.14}{9.49}\right)\left(\frac{rHR-60.10}{10.45}\right)\\
& +0.05\left(\frac{Age-44.14}{9.49}\right)\left(\frac{BMI-26.46}{3.50}\right)-0.09\left(\frac{Age-44.14}{9.49}\right)\left(\frac{SBP-120.58}{12.70}\right)\\
& +0.04\left(\frac{Age-44.14}{9.49}\right)\left(\frac{DBP-81.09}{9.32}\right)+0.07\left(\frac{Wt-85.04}{12.84}\right)\left(\frac{Ht-179.15}{6.51}\right)\\
& -0.07\left(\frac{Ht-179.15}{6.51}\right)^2+0.04\left(\frac{Ht-179.15}{6.51}\right)\left(\frac{rHR-60.10}{10.45}\right)\\
& -0.05\left(\frac{Ht-179.15}{6.51}\right)\left(\frac{BMI-26.46}{3.50}\right)-0.01\left(\frac{Ht-179.15}{6.51}\right)\left(\frac{SBP-120.58}{12.70}\right)\\
& +0.11\left(\frac{rHR-60.10}{10.45}\right)^2+0.13\left(\frac{rHR-60.10}{10.45}\right)\left(\frac{BMI-26.46}{3.50}\right)\\
& +0.03\left(\frac{rHR-60.10}{10.45}\right)\left(\frac{SBP-120.58}{12.70}\right)-0.01\left(\frac{rHR-60.10}{10.45}\right)\left(\frac{DBP-81.09}{9.32}\right)\\
& -0.09\left(\frac{BMI-26.46}{3.50}\right)\left(\frac{SBP-120.58}{12.70}\right)+0.04\left(\frac{BMI-26.46}{3.50}\right)\left(\frac{DBP-81.09}{9.32}\right)\\
& +0.02\left(\frac{SBP-120.58}{12.70}\right)^2-0.06\left(\frac{SBP-120.58}{12.70}\right)\left(\frac{DBP-81.09}{9.32}\right)+0.01\left(\frac{DBP-81.09}{9.32}\right)^2\\
& +0.88(1-Smoking)+11.29
\end{aligned}
$$

Women's equation

$$
\begin{aligned}
METs = {} & -0.71\left(\frac{Age-44.11}{10.17}\right)+0.09\left(\frac{Ht-164.62}{5.96}\right)-0.63\left(\frac{rHR-64.02}{10.11}\right)-0.75\left(\frac{BMI-23.31}{3.68}\right)\\
& +0.17\left(\frac{SBP-112.42}{13.94}\right)-0.10\left(\frac{Age-44.11}{10.17}\right)^2-0.07\left(\frac{Age-44.11}{10.17}\right)\left(\frac{Ht-164.62}{5.96}\right)\\
& +0.08\left(\frac{Age-44.11}{10.17}\right)\left(\frac{rHR-64.02}{10.11}\right)+0.10\left(\frac{Age-44.11}{10.17}\right)\left(\frac{BMI-23.31}{3.68}\right)\\
& -0.05\left(\frac{Age-44.11}{10.17}\right)\left(\frac{SBP-112.42}{13.94}\right)+0.02\left(\frac{Wt-63.21}{10.78}\right)\left(\frac{rHR-64.02}{10.11}\right)\\
& +0.02\left(\frac{Ht-164.62}{5.96}\right)^2-0.01\left(\frac{Ht-164.62}{5.96}\right)\left(\frac{BMI-23.31}{3.68}\right)\\
& +0.07\left(\frac{Ht-164.62}{5.96}\right)\left(\frac{SBP-112.42}{13.94}\right)+0.10\left(\frac{rHR-64.02}{10.11}\right)^2\\
& +0.05\left(\frac{rHR-64.02}{10.11}\right)\left(\frac{BMI-23.31}{3.68}\right)-0.02\left(\frac{rHR-64.02}{10.11}\right)\left(\frac{DBP-76.04}{9.33}\right)\\
& +0.01\left(\frac{BMI-23.31}{3.68}\right)^2-0.06\left(\frac{BMI-23.31}{3.68}\right)\left(\frac{DBP-76.04}{9.33}\right)\\
& -0.03\left(\frac{SBP-112.42}{13.94}\right)\left(\frac{DBP-76.04}{9.33}\right)-0.01\left(\frac{DBP-76.04}{9.33}\right)^2+0.77(1-Smoking)\\
& +9.07
\end{aligned}
$$
